# Differential effect of human ivermectin treatment on blood feeding *Anopheles gambiae* and *Culex quinquefasciatus*

**DOI:** 10.1186/s13071-015-0735-3

**Published:** 2015-02-27

**Authors:** Yahya A Derua, William N Kisinza, Paul E Simonsen

**Affiliations:** National Institute for Medical Research, Amani Research Centre, P.O. Box 81, Muheza, Tanga Tanzania; Department of Veterinary Disease Biology, Faculty of Health and Medical Sciences, University of Copenhagen, Dyrlægevej 100, 1870 Frederiksberg, Copenhagen, Denmark

**Keywords:** Ivermectin, *Anopheles gambiae*, *Culex quinquefasciatus*, Tanzania

## Abstract

**Background:**

Widespread and large scale use of ivermectin in humans and domestic animals can have unexpected effects on non-target organisms. As a search for a possible explanation for an observed longitudinal decline in density of anopheline vector mosquitoes, but not in *Culex quinquefasciatus,* in an area of north-eastern Tanzania which has been exposed to ivermectin mass drug administration, this study assessed and compared the effect of human ivermectin treatment on blood feeding *Anopheles gambiae* and *Cx. quinquefasciatus*.

**Methods:**

Consenting adult volunteers were randomized into two groups to receive either ivermectin or placebo. Twenty four hours after treatment, one volunteer from each group was concurrently exposed to 50 laboratory reared *An. gambiae* on one arm and 50 laboratory reared *Cx. quinquefasciatus* on the other arm for 15–30 minutes. Engorged mosquitoes were maintained on 10% glucose solution for 12 days and observed for survival and fecundity. The experiment was repeated 15 times.

**Results:**

Two days after the blood meals, nearly half (average 47.7% for the 15 experiments) of the blood fed *An. gambiae* in the ivermectin group had died while almost all in the placebo group were alive (97.2%), and the difference in survival between these two groups continued to widen on the following days. There was no clear effect of ivermectin on *Cx. quinquefasciatus,* which had high survival in both ivermectin and placebo group on day 2 (95.7% and 98.4%, respectively) as well as on the following days. Ivermectin completely inhibited egg laying in *An. gambiae,* while egg laying and subsequent development of immature stages appeared normal in the other three groups.

**Conclusion:**

Blood meals taken on ivermectin treated volunteers significantly reduced survival and halted fecundity of *An. gambiae* but had only limited or no effect on *Cx. quinquefasciatus*. The result suggests that widespread use of ivermectin may have contributed to the observed decline in density of *An. gambiae,* without similar decrease in *Cx. quinquefasciatus*, in north-eastern Tanzania.

## Background

Ivermectin, a macrocyclic lactone derived as a fermentation product from the soil bacteria *Streptomyces avermilitis* [1], is widely used globally in human and veterinary medicine as a drug for treatment and control of nematode and ecto-parasitic infections. It is currently also used in large scale programs to eliminate human onchocerciasis and lymphatic filariasis in many countries in sub-Saharan Africa, through yearly application of community-wide ivermectin mass drug administration (MDA) [2]. In concordance with the extensive scale-up and use of ivermectin in recent years, its potential effect on non-target organisms, including many species of free-living arthropods, has been increasingly documented [3]. This includes its effect on dung beetles being exposed to ivermectin when feeding on faecal matter from treated cattle, as well as on freshwater stages of insects exposed to ivermectin excreted into their environment.

Of recent, it has moreover been documented that female *Anopheles gambiae* mosquitoes have a much higher mortality if they feed on humans shortly after treatment with ivermectin than if they feed on untreated humans [4]. Laboratory studies using membrane feeding of adult mosquitoes have confirmed this effect of ivermectin, when used in concentrations equaling those found in treated humans, in female *An. gambiae* whereas no effect was seen in female *Aedes aegypti* [5,6]. In line with this, field studies have indicated that human MDA with ivermectin may have resulted in a temporary significant reduction in malaria transmission, most likely due to its effect on the anopheline vectors [7-10]. This has led to the suggestion that human MDA with ivermectin could be used as a supplement measure for malaria transmission control [9-12].

In Tanzania, *An. gambiae* and *An. funestus* are the major vectors of human malaria, and these two mosquito species together with *Cx. quinquefasciatus* are also the vectors of lymphatic filariasis. A longitudinal study conducted in north-eastern Tanzania has documented a dramatic decline in anopheline mosquito densities during the past decade, whereas no decline has been seen in densities of *Cx. quinquefasciatus* [13,14]. The cause of these changes, which have important implications for the transmission and control of malaria and lymphatic filariasis in the area, remains somewhat unclear, and could not immediately be related to change in rainfall pattern or major application of mosquito control interventions [14]. In fact, insecticide treated bed-nets (ITNs) were only distributed on a large scale after the major decline in anopheline population densities occurred. As MDA with ivermectin has been widely applied in the area for control of onchocerciasis and lymphatic filariasis [13,15], the present study assessed and compared the survival and fecundity of *An. gambiae* and *Cx. quinquefasciatus* mosquitoes after taking a blood meal on humans treated with ivermectin.

## Methods

### Mosquitoes

*An. gambiae* sensu stricto (a colony originating from Kisumu, Kenya) and *Cx. quinquefasciatus* (a colony originating from Arusha, Tanzania) mosquitoes which had been maintained for several generations at the insectary of the National Institute for Medical Research, Amani Research Centre, in Tanga Region of Tanzania, were used in the feeding experiments. They were raised using recommended standard mosquito rearing techniques [16]. Adult mosquitoes for feeding experiments were kept in cages and supplied with cotton pads soaked in 10% glucose solution until they reached an age of 3 days. They were then starved for 6–8 hours before being fed on the human volunteers.

### Experimental drugs

Ivermectin tablets (Mectzan®, Laboratoires Merck Sharp & Dohme-Chibret, Paris, France) were generously supplied by the Regional Neglected Tropical Diseases Control Programme in Tanga. This programme also provided height guides for estimation of dosage according to procedures used during MDAs (150–200 μg/kg body weight). Multivitamin tablets (Laboratory & Allied Ltd, Nairobi, Kenya) were used as placebo at a dose of 2 tablets per person.

### Selection and treatment of volunteers

Amani Research Centre staff and community members living near the Amani Research Centre premises were invited to participate in the study. A total of 30 adult volunteers, who gave written informed consent, were recruited for the feeding experiments. Volunteers were randomly assigned to either an ivermectin (n = 15) or a placebo (n = 15) treatment group. This assignment was blinded to the volunteers, to those who provided them the drugs as well as to those who handled the mosquito feeding and subsequently observed mosquito survival. The volunteers were thereafter arranged in 15 pairs, each with an ivermectin and a placebo group member, to participate in 15 mosquito feeding experiments on 15 different days. Each experiment started by requesting the pair of volunteers to swallow their respective assigned drug in the evening at 18:00 hours. They were then requested to return 24 hours later for mosquito feeding.

### Mosquito feeding and mortality scoring

At the time of mosquito feeding, 50 *An. gambiae* and 50 *Cx. quinquefasciatus* maintained in cages were concurrently allowed to feed on exposed arms of each volunteer for 15 to 30 minutes (until no more mosquitoes showed interest in feeding on the bait). Each volunteer was instructed to insert the right arm in the cage containing 50 *An. gambiae* and the left arm in the cage containing 50 *Cx. quinquefasciatus* mosquitoes. The mosquito cages were labeled with codes identifying the feeder, the treatment given and the species of mosquito. Thus, feeding experiments were carried out with two volunteers at a time (ivermectin and placebo group) and repeated 15 times using new volunteers and new batches of mosquitoes. Following the feeding, unfed mosquitoes were removed from the cage using an aspirator. Fully engorged mosquitoes were maintained on 10% glucose solution (soaked onto cotton wool) and observed for mortality every 24 hours for 12 days. On each day, dead mosquitoes were counted and removed from the cages.

The test mosquitoes were provided with egg laying substrate to monitor their egg laying ability three days (72 hours) after the blood meal. *An. gambiae* mosquitoes were provided with a wet filter paper fitted in a Petri-dish and placed inside the maintenance cage with surviving *An. gambiae* mosquitoes. For *Cx. quinquefasciatus*, a plastic larvae-rearing pan containing water was placed in the cage with survivors of this species. These devices were left in place for three days and then transferred to a larvae-rearing room for incubation and egg hatching. Eggs were not counted. Upon hatching, the offspring were reared to adult mosquitoes following routine procedures.

### Data analysis

Data were entered in Excel and subsequently analyzed by SPSS. Proportions were compared by Pearson’s Chi-square test and continuous variables by Student’s *t*-test. P-values < 0.05 were considered statistically significant.

### Ethics

Ethical approval for this study was obtained from the Medical Research Coordinating Committee of the National Institute for Medical Research, Tanzania (Ref: NIMR/HQ/R.8a/Vol. IX/1554). Written informed consent was obtained from all volunteers before they participated in the study. Mosquito feeding experiments were conducted using insectary reared mosquitoes free from any infection. The study coincided with the timing of ivermectin based MDA in the area, and volunteers who took ivermectin were informed not to take the ivermectin distributed in the communities in 2013. Volunteers who took multivitamin were encouraged to participate in the 2013 MDA. The volunteers were also told that they would experience minor discomfort and itching during mosquito feeding, resembling their usual experience with mosquito bites in north-eastern Tanzania.

## Results

A total of 30 adult volunteers (25 males, 5 females; mean age 32.4 years; age range 19–50 years) were assigned to either the ivermectin (n = 15) or the placebo (n = 15) treatment group. Fifteen consecutive mosquito feeding experiments were conducted. In each experiment, one volunteer from each treatment group fed *An. gambiae* and *Cx. quinquefasciatus* simultaneously (each volunteer arm with different species). Of the 1500 *An. gambiae* and 1500 *Cx. quinquefasciatus* exposed to the feeding baits, 1461 (97.4%) and 947 (63.1%), respectively, fed (Table 1). Mosquitoes were moreover attracted differently by individual volunteers such that for *An. gambiae* a mean of 48.7 mosquitoes fed (range 36–50) while for *Cx. quinquefasciatus* a mean of 31.6 mosquitoes fed (range 8–50) in the 30 feeding sessions (2 sessions in each of the 15 experiments). However, there was no significant difference in overall percentage of mosquitoes that fed in the ivermectin or the placebo group, neither for *An. gambiae* (97.6% vs. 97.1%, respectively; p = 0.75) nor for *Cx. quinquefasciatus* (63.7% vs. 62.5%, respectively; p = 0.67).

**Table 1:** **Overview of mosquitoes exposed and mosquitoes that fed in the two treatment groups**

**Treatment group**	**Mosquito species**	**No. exposed**	**No. fed (%)**
Ivermectin	*An. gambiae*	750	732 (97.6)
	*Cx. quinquefasciatus*	750	478 (63.7)
	All	1500	1210 (80.7)
Placebo	*An. gambiae*	750	729 (97.1)
	*Cx. quinquefasciatus*	750	469 (62.5)
	All	1500	1198 (79.9)

The mean number (and range) of live blood fed mosquitoes that survived in each of the four experimental groups during the 12 days are shown in Table 2. For *An. gambiae*, blood fed mosquitoes died considerably faster in the ivermectin than the placebo group. Already on day 2, nearly half of the blood fed *An. gambiae* in the ivermectin group had died while those in the placebo group appeared largely unaffected. The gap between the two groups in mean number of surviving mosquitoes widened from day to day, and on day 6 the mean number was only 10% of starting value for the ivermectin group while it was 91% for the placebo group. Statistical analysis showed that from day 2 onwards the mean number of live mosquitoes was significantly lower in the ivermectin group than in the placebo group (p < 0.001 for all days). In the later period of the experiments (especially after day 8) some mortality also became evident in the placebo group, possibly mainly due to increasing mosquito age.

**Table 2:** **Mean number (range) of live blood fed mosquitoes for the 15 experiments as recorded on each day in the four experimental groups**

**Day**	**Mean no. (range) of** ***An. gambiae***			**Mean no. (range) of** ***Cx. quinquefasciatus***		
	**Ivermectin group**	**Placebo group**	p-value*	**Ivermectin group**	**Placebo group**	p-value*
0	48.8 (36–50)	48.6 (40–50)	NS	31.9 (13–50)	31.3 (8–50)	NS
1	45.5 (28–50)	48.3 (40–50)	NS	31.1 (11–50)	31.2 (8–50)	NS
2	25.4 (9–45)	47.2 (40–50)	<0.001	30.7 (11–50)	30.9 (7–50)	NS
3	16.7 (0–37)	46.4 (40–50)	<0.001	30.1 (11–49)	30.7 (7–50)	NS
4	10.4 (0–18)	45.7 (40–50)	<0.001	29.9 (11–49)	30.7 (7–50)	NS
5	6.5 (0–14)	44.9 (39–49)	<0.001	29.5 (11–49)	30.3 (7–50)	NS
6	5.0 (0–14)	44.3 (39–49)	<0.001	29.1 (10–49)	29.6 (7–50)	NS
7	2.9 (0–10)	42.3 (38–49)	<0.001	28.9 (10–49)	29.3 (7–50)	NS
8	2.4 (0–10)	33.3 (20–49)	<0.001	27.9 (10–48)	27.9 (6–50)	NS
9	1.7 (0–5)	27.8 (5–43)	<0.001	27.5 (9–48)	27.5 (6–50)	NS
10	1.5 (0–4)	23.3 (1–39)	<0.001	26.5 (9–48)	27.1 (5–50)	NS
11	1.3 (0–4)	21.3 (0–36)	< 0.001	25.0 (9–48)	26.1 (5–48)	NS
12	0.8 (0–2)	16.0 (0–36)	< 0.001	20.5 (0–48)	21.1 (0–48)	NS

*Student’s *t*-test; NS = not significant.

By contrast, no clear effect of ivermectin was observed on the mean number of blood fed *Cx. quinquefasciatus* (Table 2)*,* and on none of the experimental days was there a statistically significant difference between the mean number in the ivermectin and the placebo group (p > 0.05 for all days). There was also a gradual but slight decrease in mean number of mosquitoes in both treatment groups (most noticeable after day 8), which possibly was mainly due to increasing mosquito age.

**Figure 1:**
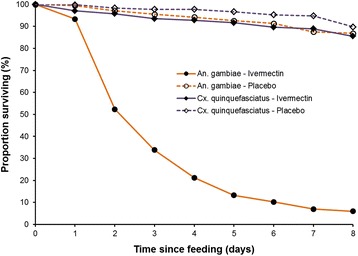
**Survival of** ***Anopheles gambiae*** **and** ***Culex quinquefasciatus*** **after taking a blood meal on human volunteers who had ingested ivermectin (150–200 μg/kg) or placebo.** Graphs show mean of % survival in the individual experiments for the four groups during the first 8 days. 15 experiments were carried out, but data were excluded from both ivermectin and placebo group of the same mosquito species when the mortality in the placebo group exceeded 20%. Thus, for the two *An. gambiae* groups, data on days 7 and 8 are based on 13 and 6 experiments, while for the two *Cx. quinquefasciatus* groups, data on days 6, 7 and 8 are all based on 13 experiments (see also Table 3).

As the number of mosquitoes feeding varied between experiments, the percent survival should rightly be calculated separately for each experiment and group, and the mean survival in groups then calculated on the basis of survival in the individual experiments. This has been done in Table 3, where moreover data have been excluded from both ivermectin and placebo groups when the mortality in the placebo group exceeded 20%. This gives an even more striking impression of the difference in *An. gambiae* survival between the ivermectin and the placebo group, and of the lack of difference between these groups in *Cx. quinquefasciatus*. The mean survival in the four experimental groups until day 8 (when mortality in the placebo groups was minimal in most experiments) is moreover shown in Figure 1.

**Table 3:** **Mosquito survival on each day of the experiment in the four groups**

**Day**	***An. gambiae***			***Cx. quinquefasciatus***		
	**No. experiments included**	Mean % survival in ivermectin group*	Mean % survival in placebo group*	**No. experiments included**	Mean % survival in ivermectin group*	Mean % survival in placebo group*
0	15	100.0	100.0	15	100.0	100.0
1	15	93.4	99.5	15	97.1	99.9
2	15	52.3	97.2	15	95.7	98.4
3	15	33.8	95.6	15	93.5	97.8
4	15	21.2	94.2	15	92.8	97.8
5	15	13.3	92.6	15	91.7	96.7
6	15	10.3	91.3	13	89.6	95.3
7	13	7.0	87.6	13	88.9	94.9
8	6	6.0	86.8	13	85.5	89.8
9	2	5.0	88.0	13	83.6	88.0
10	1	0.0	80.0	13	80.4	86.4
11	1	0.0	80.0	12	75.1	85.7
12	1	0.0	80.0	9	80.3	86.4

Calculated as mean of % survival in the individual experiments. For each of the two species, results from experiments were excluded when mortality in the placebo group exceeded 20%.

*Mean for included experiments.

Three days after the feeding, a total of 247 and 697 surviving *An. gambiae* and 447 and 459 surviving *Cx. quinquefasciatus* in the ivermectin and placebo groups, respectively, were provided with egg laying substrates. None of the *An. gambiae* in the ivermectin group laid eggs, although the majority spent long periods on the laying substrate, and eventually some were recovered dead on the substrate. Surviving *An. gambiae* in the placebo group as well as *Cx. quinquefasciatus* in both groups laid eggs. The eggs hatched and larvae were subsequently reared to adult mosquitoes.

## Discussion

Ivermectin has a broad spectrum of activity against helminth and ecto-parasite infections and its widespread use in human and veterinary medicine has been linked with potential effects on non-target organisms [3]. Ivermectin based MDA to humans for control and elimination of onchocerciasis and lymphatic filariasis has been reported to reduce survival of *An. gambiae* mosquitoes and thereby also to affect malaria transmission [7,8,10]. As a result, ivermectin has recently been considered a drug with potential in integrated malaria control operations [8,10-12]. MDA with ivermectin is also well known to have a beneficial effect on human infections with ectoparasites, such as scabies and lice [2]. In north-eastern Tanzania, where ivermectin based MDA has been in place for more than a decade, a significant decline in overall malaria vector density has been recorded. During the same period, no decline (rather an increase) in density of *Cx. quinquefasciatus* was noted [13,14]. The observed changes could not explicitly be explained by change in rainfall pattern or mosquito control interventions. Thus, this study was a sequel of an effort to indirectly assess whether ivermectin based MDAs could have contributed to causing the observed changes in mosquito population densities in the study area.

Using insectary reared *An. gambiae* and *Cx. quinquefasciatus*, 15 mosquito feeding experiments were conducted to compare the survival and fecundity of these vectors when fed on human hosts recently treated with ivermectin or a placebo. The two species of mosquitoes had different feeding efficiency, with 97.4% and 63.1%, respectively, taking a blood meal. The lower efficiency of *Cx. quinquefasciatus* was probably due to its preference for bird feeding [17] whereas *An. gambiae* is known to be strongly anthropophilic [18]. For both species, the number of fed mosquitoes moreover varied between the volunteers. Such differential human attractiveness to mosquitoes is a common phenomenon [19]. In some of the experiments, an increase was seen in mortality in the control group in the later period of the study, probably due to older age of mosquitoes and/or adverse environmental factors. For this reason, experimental days where control group mortality exceeded 20% were excluded when comparing mosquito survival in the ivermectin and control group for the same species.

The study confirmed findings from previous studies, in that, at a dosage similar to that used in MDAs, ivermectin considerably reduced the survival of blood feeding *An. gambiae* [4,7-12,20]. Thus, after 2 days nearly half (47.7%) of the blood fed *An. gambiae* in the ivermectin group had died while almost all in the placebo group were alive, and the difference in survival between these two groups continued to widen as the experiment progressed. On the other hand, ivermectin had only minimal or no effect on the survival of *Cx. quinquefasciatus*. Chandre and Hougard [21] similarly recorded no effect on survival of *Cx. quinquefasciatus* exposed to a blood meal from humans treated with a therapeutic dose of ivermectin. In future studies it will be interesting also to feed mosquitoes on volunteers at different time intervals after ivermectin treatment, to assess the time-course of the drug effect on the mosquitoes.

In addition to considerably reduce their survival, ivermectin in the blood meal caused complete inhibition of fecundity in the exposed *An. gambiae*. Thus, among the *An. gambiae* that managed to survive for 3 days post blood meal, none were capable of laying eggs. By contrast, the *An. gambiae* in the placebo group, and the *Cx. quinquefasciatus* in both groups laid eggs. The eggs were incubated, hatched and larvae were successfully reared to adult mosquitoes. The eggs were not counted, though, to compare if there was any difference in numbers laid by *Cx. quinquefasciatus* in the ivermectin and placebo group, or in their subsequent development. This would be worthwhile to investigate further, as in *Ae. aegypti* mosquitoes it has been shown that although an ivermectin containing blood meal did not affect survival, it caused a significant decline in fecundity [22].

The fact that *Cx. quinquefasciatus* has a slightly lower feeding capacity on humans, combined with their apparent inherent tolerance to ivermectin in blood meals, put them at a much reduced risk of being affected by ivermectin based MDA when compared to *An. gambiae*. These factors, combined with evidence of increasing proliferation and colonization of more rural areas by *Cx. quinquefasciatus* [23,24] and their high level of insecticide tolerance [17,25], might explain the stable density of this species in the areas with declining anopheline density in north-eastern Tanzania [14]. The increasing significance of *Cx. quinquefasciatus* as a vector of lymphatic filariasis in north-eastern Tanzania, as compared to the previously most important anopheline vectors [13,26], calls for more research on how to control this vector.

## Conclusions

The study showed that mosquito blood meals taken from humans recently treated with ivermectin at a dosage similar to that used in MDA for control of filarial infections have a markedly different effect on survival and fecundity of *An. gambiae* and *Cx. quinquefasciatus*. Thus, while ivermectin treatment considerably reduced survival and fecundity in the former, no obvious effect was noted in the latter species. The findings suggest that the recently observed decline in density of *An. gambiae*, but not *Cx. quinquefasciatus*, in north-eastern Tanzania might at least partly be attributed to the widespread use of ivermectin for control of parasitic infections in humans and domestic animals in the area.
